# Visualizing the distribution of flavonoids in litchi (*Litchi chinenis*) seeds through matrix-assisted laser desorption/ionization mass spectrometry imaging

**DOI:** 10.3389/fpls.2023.1144449

**Published:** 2023-02-24

**Authors:** Yukun Liu, Xiaofei Nie, Jilong Wang, Zhenqi Zhao, Zhimei Wang, Fang Ju

**Affiliations:** ^1^ Department of Breast Surgery, Breast Disease Center, Affiliated Qingdao Central Hospital, Qingdao University, Qingdao, China; ^2^ Department of Oncology, Affiliated Qingdao Central Hospital, Qingdao University, Qingdao, China; ^3^ Department of Acupuncture and Moxibustion, Affiliated Qingdao Central Hospital, Qingdao University, Qingdao, China; ^4^ Department of Radiology, Affiliated Qingdao Central Hospital, Qingdao University, Qingdao, China; ^5^ Department of Gynecological Neoplasms, Affiliated Qingdao Central Hospital, Qingdao University, Qingdao, China

**Keywords:** litchi seed, flavonoid, matrix-assisted laser desorption/ionization, mass spectrometry imaging, spatial distribution

## Abstract

Flavonoids are one of the most important bioactive components in litchi (*Litchi chinensis* Sonn.) seeds and have broad-spectrum antiviral and antitumor activities. Litchi seeds have been shown to inhibit the proliferation of cancer cells and induce apoptosis, particularly effective against breast and liver cancers. Elucidating the distribution of flavonoids is important for understanding their physiological and biochemical functions and facilitating their efficient extraction and utilization. However, the spatial distribution patterns and expression states of flavonoids in litchi seeds remain unclear. Herein, matrix-assisted laser desorption/ionization mass spectrometry imaging (MALDI-MSI) was used for *in situ* detection and imaging of the distribution of flavonoids in litchi seed tissue sections for the first time. Fifteen flavonoid ion signals, including liquiritigenin, apigenin, naringenin, luteolin, dihydrokaempferol, daidzein, quercetin, taxifolin, kaempferol, isorhamnetin, myricetin, catechin, quercetin 3-β-d-glucoside, baicalin, and rutin, were successfully detected and imaged *in situ* through MALDI-MSI in the positive ion mode using 2-mercaptobenzothiazole as a matrix. The results clearly showed the heterogeneous distribution of flavonoids, indicating the potential of litchi seeds for flavonoid compound extraction. MALDI-MS-based multi-imaging enhanced the visualization of spatial distribution and expression states of flavonoids. Thus, apart from improving our understanding of the spatial distribution of flavonoids in litchi seeds, our findings also facilitate the development of MALDI-MSI-based metabolomics as a novel effective molecular imaging tool for evaluating the spatial distribution of endogenous compounds.

## Introduction

Litchi (*Litchi chinensis* Sonn.; order Sapindales, family Sapindaceae), also known as Lizhi, Danli, and Liguo, is a subtropical fruit tree with a cultivation history in China of more than 2,300 years (Hu et al., 2021). It is the only species of *Litchi* (Yao et al., 2021). Litchi is an important fruit crop in southern China and is planted on more than 550,000 ha with an annual output of more than 2.2 million tons (Hu et al., 2021). The cultivation area and output of litchi in China account for more than 60% of global production (Li et al., 2020). Litchi seeds are a major product, but only a small portion is processed for biological utilization, and many litchi seeds are discarded as waste. The abandonment of fruit seed residues is not only a considerable problem for the environment but also a waste of global resources. Litchi seeds are rich in various bioactive compounds, such as flavonoids, saponins, volatile oils, polyols, alkaloids, steroids, coumarins, fatty acids, amino acids, and sugars (Dong et al., 2019; Punia and Kumar, 2021), resulting in a variety of biological functions, including antiviral and anti-oxidation activities, reducing the degree of liver damage and lowering blood glucose levels (Choi et al., 2017; Dong et al., 2019; Punia and Kumar, 2021). Accumulating evidence has confirmed the antitumor/anticancer effects of litchi seed extracts (Emanuele et al., 2017; Tang et al., 2018; Zhao et al., 2020).

Flavonoids are polyphenolic compounds and endogenous bioactive components, which act as secondary metabolites with extensive pharmacological activities. Flavonoids exert important pharmacological properties, including cardioprotective, anticancer, anti-inflammatory, and anti-allergic activities (Maleki et al., 2019; Ciumărnean et al., 2020; Liskova et al., 2021; Rakha et al., 2022). Regarding anticancer activity, many preclinical studies indicated the antiproliferative effects of flavonoids on lung (Berk et al., 2022), prostate (Vue et al., 2016), colorectal (Park et al., 2012; Li et al., 2018b), and breast (Pan et al., 2012) cancers. Furthermore, flavonoids have anticancer effects on breast tumors through multiple mechanisms (Martinez-Perez et al., 2014; Magne Nde et al., 2015; Zhang et al., 2018; Sudhakaran et al., 2019). Flavonoids can inhibit procarcinogen bioactivation and estrogen-producing and estrogen-metabolizing enzymes (Surichan et al., 2012; Miron et al., 2017), as well as breast cancer resistance protein (BCRP) (Fan et al., 2019). Administering flavonoids could inhibit inflammation, proliferation, tumor growth, and metastasis (Peluso et al., 2013; Khan et al., 2021; Guo et al., 2022). Although many studies have shown the pharmacological effects of flavonoids widely distributed in litchi seeds, almost all such studies were based on the extraction, enrichment, and separation of bioactive components, and few have focused on the spatial distribution and expression states of flavonoids. In fact, the precise reveal of the distribution of these flavonoids in litchi seeds is important for understanding the physiological and biochemical functions of these compounds and facilitating their extraction and utilization.

Matrix-assisted laser desorption/ionization mass spectrometry imaging (MALDI-MSI) has emerged as a molecular-imaging tool for simultaneously detecting and characterizing the spatial distribution and relative abundance of endogenous and exogenous compounds, such as lipids, proteins, metabolites, peptides, and drugs (Van De Plas et al., 2015; Qin et al., 2018; Piehowski et al., 2020). Although MALDI‐MSI has been used in plant science with endogenous molecular profiling to determine the spatial distribution of small molecules in plant tissues (Zaima et al., 2010; Taira et al., 2015; Huang et al., 2016; Li et al., 2018a), to the best of our knowledge, no previous study has utilized MALDI-MSI to characterize the spatial distribution of flavonoids in litchi seeds.

This study is the first to use MALDI-MSI for the *in situ* detection and imaging of flavonoids in litchi seed tissues. The results clearly showed the heterogeneous distribution of flavonoids in litchi seeds, indicating the potential of litchi seeds as a source for flavonoid extraction. MALDI-MS-based multi-imaging enhanced the visualization of spatial distribution and expression states of flavonoids. Our findings provide insights into the spatial distribution of flavonoids in litchi seeds and support the development of MALDI-MSI-based metabolomics as an appealing and credible molecular imaging technique for evaluating the spatial distribution of endogenous compounds.

## Materials and methods

### Materials and reagents

Fresh litchi fruit was collected from the Yongfuda litchi orchard (Haikou, Hainan, China) in June 2022. Haikou is located on Hainan Island in China. It has a typical tropical marine climate and annual sunshine duration of over 2,000 h. The climate is humid, the temperature rises fast, and the average annual precipitation is approximately 260 mm. The Yongfuda litchi orchard is located in a volcanic rock soil planting area. Once harvested, the peel and flesh of the litchi were immediately removed, and the litchi seeds were flash-frozen with liquid nitrogen by slow immersion to prevent seed shattering and endogenous compound changes. The commonly used MALDI matrix, 2-mercaptobenzothiazole (2-MBT), was obtained from Sigma-Aldrich (St. Louis, MO, USA). Amino acid and oligopeptide standards, including His, Gly-Gly-Leu (tripeptide), Ala-His-Lys (tripeptide), Leu-Leu-Tyr (tripeptide), and Arg-Gly-Asp-dTyr-Lys (pentapeptide), were purchased from Bankpeptide Biological Technology Co., Ltd. (Hefei, Anhui, China). Trifluoroacetic acid (TFA) and liquid chromatography–mass spectrometry (LC-MS)-grade methanol and ethanol were obtained from Merck & Co., Inc. (Darmstadt, Germany). Ultrapure water in the whole process of the experiments was prepared using a Millipore Milli-Q system (Bedford, MA, USA). All other reagents and chemicals were purchased from Merck, unless otherwise noted.

### Tissue sectioning

**Figure 1:**
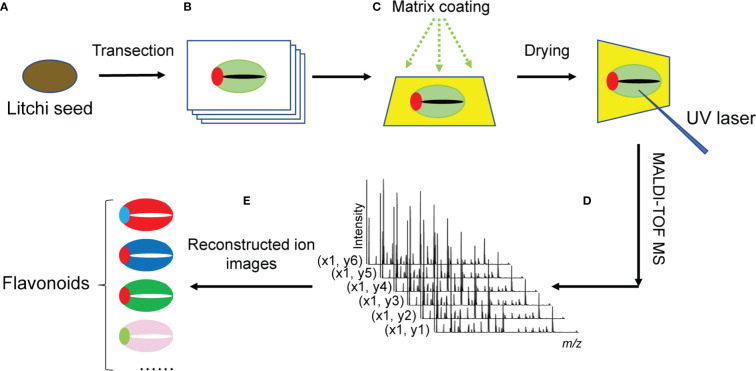
Schematic diagram of MALDI-MSI procedure for imaging flavonoids in litchi seeds. **(A)** Whole litchi seeds were used for transection into 12-μm-thick slices in a cryostat microtome. **(B)** Serial tissue sections were immediately thaw-mounted on the conductive sides of indium tin oxide (ITO)-coated microscope glass slides. Optical images of the litchi seed section were obtained using a scanner. **(C)** To assist ionization, the sections were coated with the organic matrix. **(D)** MALDI-TOF-MS was used to detect analytes *in situ* on the surface of litchi seed tissue sections. The mass spectra of ionized analytes were acquired at each detected pixel point. **(E)** MS images of analytes were reconstructed from the MS spectra obtained at each laser spot using specific imaging reconstruction software. MALDI-MSI, matrix-assisted laser desorption/ionization mass spectrometry imaging; TOF, time of fight; MS, mass spectrometry.

For tissue sectioning, a Leica CM1860 cryostat (Leica Microsystems Inc., Wetzlar, Germany) was used. The frozen litchi seeds were cryo-sectioned into 12-μm-thick slices at a temperature of −20°C, and then the cryo-sectioned samples were thaw-mounted instantly on the conductive indium tin oxide films of microscope glass slides purchased from Bruker Daltonics (Bremen, Germany) (**Figures 1A, B**).

### Matrix coating

After being air-dried, the serial litchi seed tissue sections were used for MALDI matrix coating (**Figure 1C**). A 2-MBT matrix solution was prepared at an optimal concentration of 15 mg/ml and dissolved in methanol/water/TFA (80:20:0.2, v/v/v). Air-dried tissue sections were coated with the 2-MBT matrix solution by a GET-Sprayer (III) (HIT Co., Ltd, Beijing, China). Briefly, the 2-MBT matrix solution 15 cycles (5 s spray, 10 s incubation, and 20 s drying time) was sprayed on the surface of the tissue sections to pre-seed a thin layer of the 2-MBT matrix. After the tissue sections were completely air-dried in a vented fume hood, the matrix solution was evenly sprayed for 50 more of the same cycles.

### Histological staining

In order to obtain the histological images of litchi seed tissue sections, a slightly modified hematoxylin and eosin staining method was carried out based on an established procedure (Casadonte and Caprioli, 2011). Briefly, the tissue sections were washed in a series of ethanol solutions (100%, 95%, 80%, and 70% aqueous ethanol; 15 s/wash). After 10-s ultrapure water washing, tissue sections were stained with hematoxylin solution for 2 min and then washed with ultrapure water and 70% and 95% aqueous ethanol for 30 s each. The eosin solution was applied for another 1 min. Then, all tissue sections were washed with 95% and 100% ethanol and xylene for 30-s dehydration.

### Optimal image acquisition

Optical images of the tissue sections were acquired using an Epson Perfection V550 photo scanner (Seiko Epson Corp, Suwa, Japan) according to previous studies (Wu et al., 2021; Shi et al., 2022).

### MALDI-MS

An Autoflex Speed MALDI time-of-fight (TOF)/TOF mass spectrometer (Bruker Daltonics) with a MALDI source equipped with a 2,000-Hz solid-state Smartbeam Nd : YAG UV laser (355 nm, Azura Laser AG, Berlin, Germany) was used for profiling and imaging (**Figure 1D**).

To acquire *in situ* (+) MS profiling data of flavonoids from the tissue sections, all mass spectra were obtained over the *m/z* range of 100 to 700, each mass spectrum included an accumulation of 50 laser scans, and each scan was amassed from 500 laser shots. Three biological replicates of the sample and three technical replicates of each biological replicate were performed for MALDI-MS data acquisition (n = 3 × 3). To acquire the images of flavonoids, a 250-μm laser raster step-size was utilized for flavonoid *in situ* detection in tissues, and each pixel (scan spot) included 300 laser shots. With the use of *FlexImaging* 4.1 (Bruker Daltonics), the three “teaching points” for the correct positioning of the solid-state UV laser (Smartbeam Nd : YAG) for spectral acquisition were marked around a tissue section using a white ink correction pen. The *m/z* values of the compound ions that can be used for external mass calibration were listed as follows: His ([M+H]^+^, *m/z* 156.0768), Gly-Gly-Leu (tripeptide, [M+H]^+^, *m/z* 246.1448), Ala-His-Lys (tripeptide, [M+H]^+^, *m/z* 355.2088), Leu-Leu-Tyr (tripeptide, [M+H]^+^, *m/z* 408.2493), and Arg-Gly-Asp-dTyr-Lys (pentapeptide, [M+H]^+^, *m/z* 620.3151). Gly-Gly-Leu (tripeptide, [M+H]^+^, *m/z* 246.1448) and Arg-Gly-Asp-dTyr-Lys (pentapeptide, [M+H]^+^, *m/z* 620.3151) ions were selected in combination with the matrix ion of 2-MBT([M+H]^+^, *m/z* 167.9942) for internal mass calibration in the cubic enhanced mode. For the MALDI-TOF-MS analysis, MS/MS spectra were acquired in collision-induced dissociation (CID) mode, and argon was used as the collision gas. The flavonoid fragment ions were acquired under the following condition: ion source 1, 19.0 kV; ion source 2, 17.4 kV; lens, 8.8 kV; reflector 1, 21.0 kV; reflector 2, 9.8 kV; and accelerating voltage, 20.0 kV. The UV laser power ranged from 65% to 90%. MS/MS spectra were recorded based on no less than 5,000 laser shots over the *m/z* range of 0 to 100 with a sampling rate of 2.00 G/s, a detector gain of 9.5×, and an electronic gain of 100 mV.

### Data analysis

For the MS profiling and MS/MS data analysis, Bruker *FlexAnalysis* 3.4 (Bruker Daltonics) was used for the preliminary viewing and processing of the mass spectra. Once the monoisotopic peak list was generated and exported, two metabolome databases (METLIN and HMDB) (Tautenhahn et al., 2012; Wishart et al., 2022) were used for the search of the detected *m/z* values of precursor ions and CID fragment ions against potential metabolite identities within an acceptable mass error of ±5 ppm. Three ion adduct forms (i.e., [M + H]^+^, [M + Na]^+^, and [M + K]^+^) were considered for the database search. For MALDI tissue imaging, Bruker *FlexImaging* 4.1 software was used for the reconstitution of the ion maps of the detected flavonoids (**Figure 1E**). For the generation of the ion images using *FlexImaging*, the mass filter width was set at 5 ppm.

### Flavonoid extraction and identification by LC-MS/MS

Flavonoids were extracted from the seeds of litchi for LC-MS/MS analysis. The details of the extraction of the flavonoids from litchi seeds and the procedure of LC-MS/MS analysis for the identification and structural confirmation of the flavonoids can be found in the **Supplementary Material**.

## Results and discussion

### Morphological characteristics of litchi seeds

**Figure 2:**
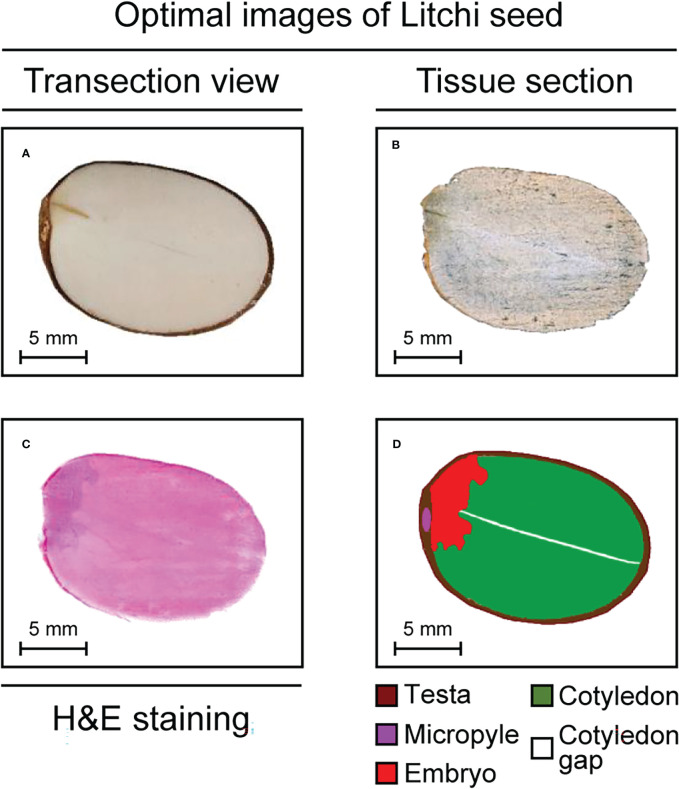
Optimal images of litchi seed tissue sections. **(A, B)** Photos of litchi seed tissue sections. **(C)** An H&E-stained litchi seed tissue section. **(D)** A cartoon of anatomical structure of litchi seed tissue section. H&E, hematoxylin and eosin.

As shown in **Figures 2A, B**, under a light microscope, the litchi seed showed the following structures: testa, micropyle, embryo, cotyledon, and cotyledon gap. Among these structures, the testa was dark coffee-colored, the embryo was brown, and the cotyledon was oyster white. In addition, a gap was observed in the middle of the cotyledon. After hematoxylin and eosin staining, litchi seeds were observed again under a light microscope (**Figure 2C**). The anatomical structure of the litchi seeds is illustrated in **Figure 2D**.

### Flavonoids detected *in situ* by MALDI-TOF-MS

**Figure 3:**
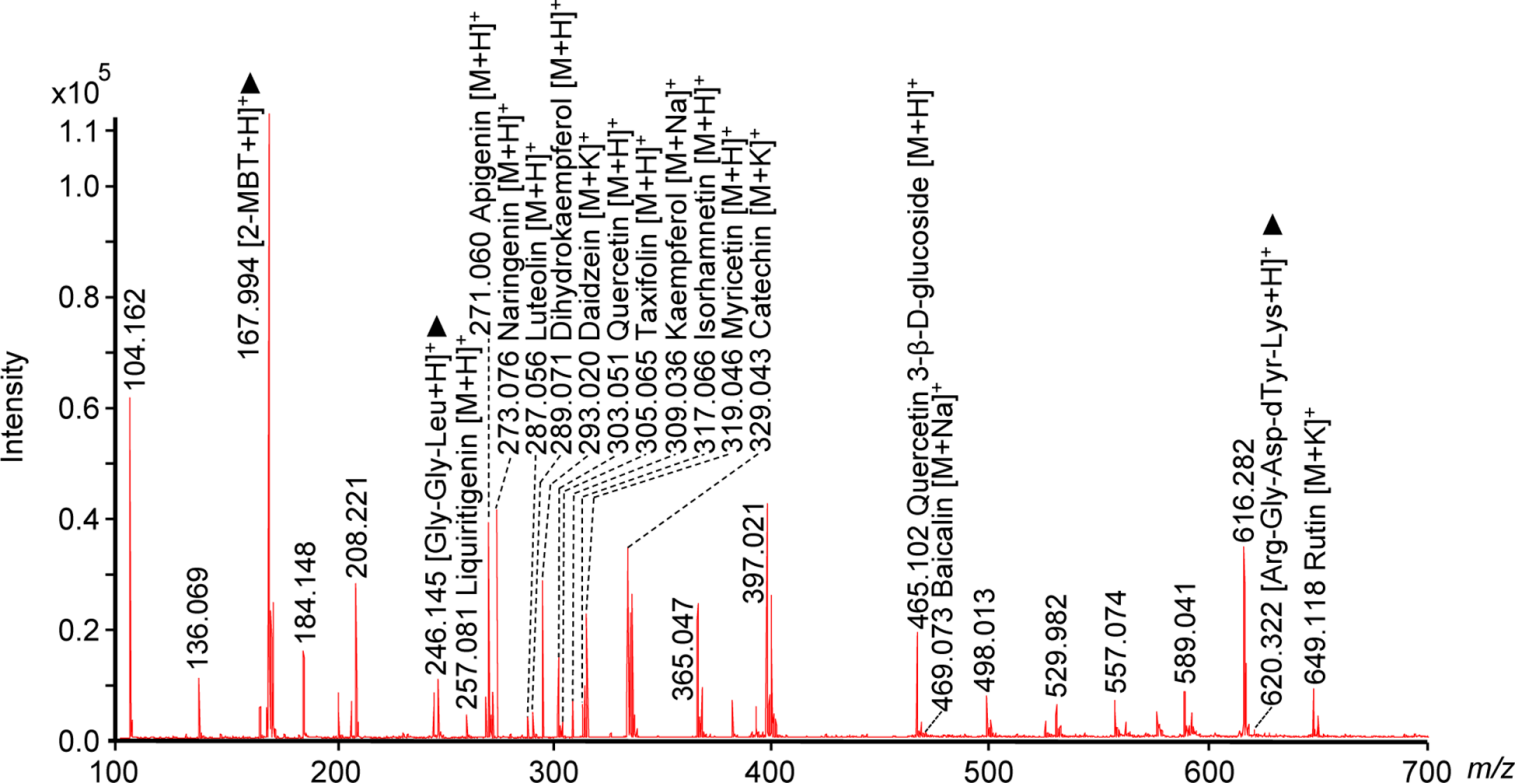
Mass spectrum of flavonoids detected *in situ* in a litchi seed tissue section using MALDI-TOF-MS and 2-MBT as the matrix in the positive ion mode. Two peptide standard ions, Gly-Gly-Leu (tripeptide, [M+H]^+^, *m/z* 246.145) and Arg-Gly-Asp-dTyr-Lys (pentapeptide, [M+H]^+^, *m/z* 620.322), and one matrix ion, 2-MBT ([M+H]^+^, *m/z* 167.994), were used as the reference peaks and are labeled with black triangle “▲”. Three biological repetitions and three technical repetitions were performed (n = 3 × 3). MALDI, matrix-assisted laser desorption/ionization; TOF, time-of-fight; MS, mass spectrometry; 2-MBT, 2-mercaptobenzothiazole.

As shown in **Figure 3**, many flavonoid-related signals were detected in the *m/z* range of 100–700. These compounds were confirmed by comparing the *m/z* values and MS/MS spectra with those obtained by LC-MS/MS (**Table 1**). According to collision-induced dissociation, 15 flavonoids compounds were identified through MALDI-TOF-MS: liquiritigenin (*m/z* 257.081, [M+H]^+^), apigenin (*m/z* 271.060, [M+H]^+^), naringenin (*m/z* 273.076, [M+H]^+^), daidzein (*m/z* 293.020, [M+Na]^+^), luteolin (*m/z* 287.056, [M+H]^+^), dihydrokaempferol (*m/z* 289.071, [M+H]^+^), catechin (*m/z* 329.043, [M+H]^+^), quercetin (*m/z* 303.051, [M+H]^+^), kaempferol (*m/z* 309.036, [M+Na]^+^), isorhamnetin (*m/z* 317.066, [M+H]^+^), myricetin (*m/z* 319.046, [M+H]^+^), quercetin 3-β-d-glucoside (*m/z* 465.102, [M+H]^+^), baicalin (*m/z* 469.073, [M+Na]^+^), rutin (*m/z* 649.118, [M+K]^+^), and taxifolin (*m/z* 305.065, [M+H]^+^).

**Table 1:** The lists of 15 detectable flavonoids in litchi seed tissue sections by MALDI-TOF-MS using 2-MBT as the matrix in the positive ion mode.

Measured *m/z*	Calculated *m/z*	Error (ppm)	Assignment			Structurally specific CID ions (*m/z*)	
			Ion form	Compound	Molecular formula	MALDI-MS/MS	LC-MS/MS
257.081	257.0808	3.1	[M+H]+	Liquiritigenin	C15H12O4	–	137.024, 147.042, 211.073, 239.068, 257.081
271.060	271.0601	0.4	[M+H]+	Apigenin	C15H10O5	119.041, 243.056, 271.060	91.051, 119.044, 153.010, 243.059, 271.060
273.076	273.0758	0.7	[M+H]+	Naringenin	C15H12O5	119.044, 147.043, 273.076	119.041, 123.038, 147.040, 153.013, 273.076
287.056	287.0550	3.5	[M+H]+	Luteolin	C15H10O6	–	135.043, 153.009, 269.044, 287.056
289.071	289.0707	1	[M+H]+	Dihydrokaempferol	C15H12O6	–	153.013, 243.062, 271.063, 289.071
293.020	293.0211	3.8	[M+K]+	Daidzein	C15H10O4	227.064, 255.062, 293.021	199.072, 227.061, 255.060, 293.020
303.051	303.0499	3.6	[M+H]+	Quercetin	C15H10O7	153.017, 201.046, 257.043, 303.050	153.014, 201.048, 229.043, 257.040, 303.051
305.065	305.0656	2	[M+H]+	Taxifolin	C15H12O7	–	123.041, 149.016, 153.012, 167.033, 231.058, 305.065
309.036	309.0370	3.2	[M+Na]+	Kaempferol	C15H10O6	–	121.022, 153.014, 165.018, 213.054, 287.051, 309.036
317.066	317.0656	1.3	[M+H]+	Isorhamnetin	C16H12O7	153.013, 229.042, 27.041, 302.027	153.012, 229.043, 274.040, 302.029, 317.066
319.046	319.0448	3.8	[M+H]+	Myricetin	C15H10O8	217.042, 245.041, 273.025, 319.045	153.011, 217.043, 245.044, 273.027, 319.046
329.043	329.0422	2.4	[M+K]+	Catechin	C15H14O6	123.043, 139.027, 165.054, 291.078	123.042, 139.026, 165.053, 291.079, 329.042
465.102	465.1028	1.7	[M+H]+	Quercetin 3-β-d-glucoside	C21H20O12	229.043, 303.042, 465.103	153.013, 229.042, 257.033, 303.044, 465.103
469.073	469.0741	2.3	[M+Na]+	Baicalin	C21H18O11	–	123.013, 271.049, 447.088, 469.07
649.118	649.1165	2.3	[M+K]+	Rutin	C27H30O16	129.053, 303.046, 465.103, 611.146, 649.118	129.054, 145.053, 147.051, 303.048, 465.102, 611.147, 649.117

Structurally specific CID ions of extracted metabolites were detected by MALDI-MS/MS or LC-MS/MS using CID. “-”: the CID ions can not be detected by MALDI-MS/MS in this work.

MALDI, matrix-assisted laser desorption/ionization; TOF, time of fight; MS, mass spectrometry; 2-MBT, 2-mercaptobenzothiazole; CID, collision-induced dissociation; LC-MS/MS, liquid chromatography–tandem mass spectrometry.

### MALDI-MS imaging of flavonoids

**Figure 4:**
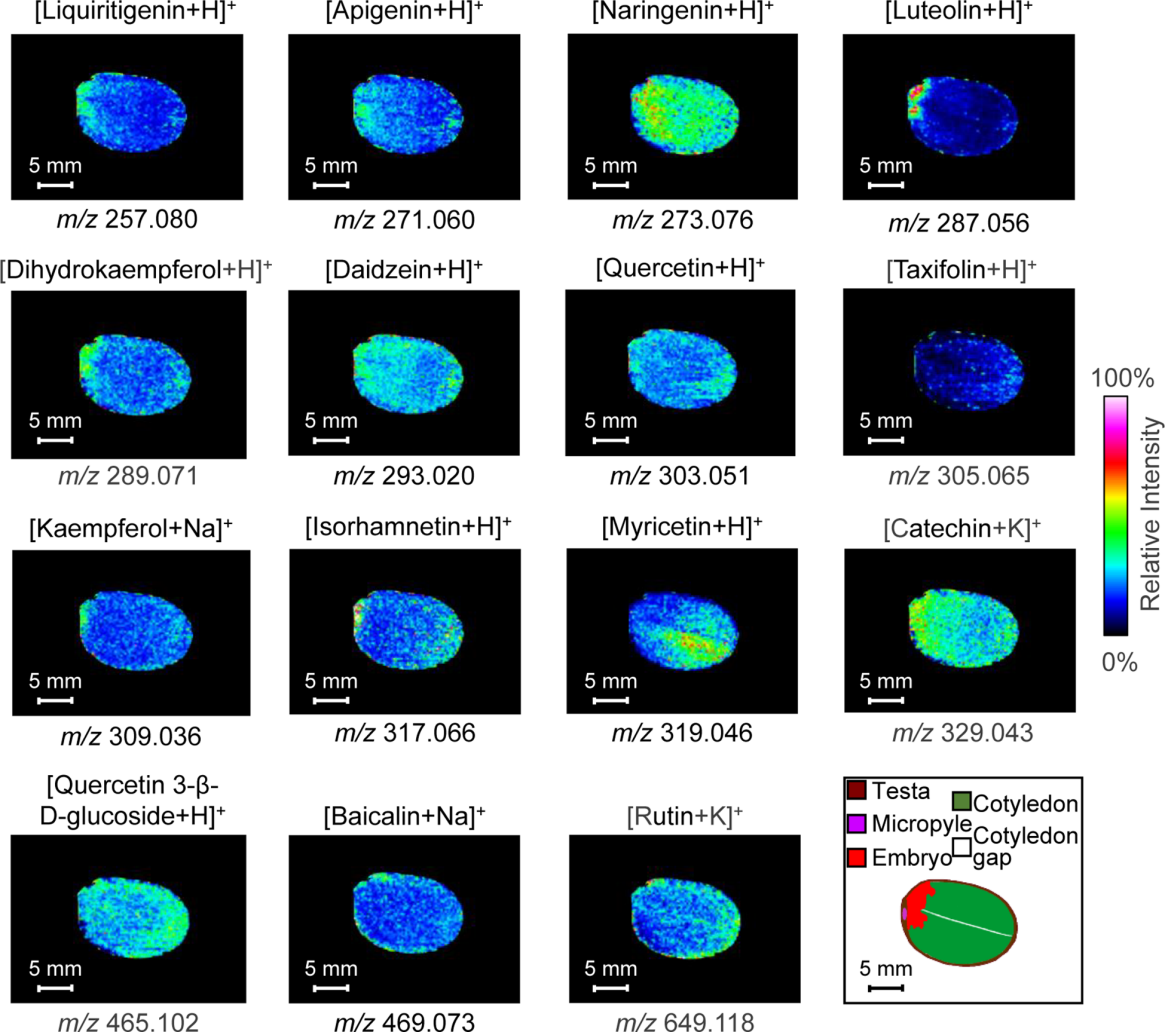
Ion images of 15 detectable flavonoids in litchi seed tissue sections from MALDI-TOF-MS using 2-MBT as the matrix in positive ion mode. MS imaging was acquired at 250-μm spatial resolution. MALDI, matrix-assisted laser desorption/ionization; TOF, time of fight; MS, mass spectrometry; 2-MBT, 2-mercaptobenzothiazole.

MALDI-MSI can provide a snapshot of the distribution of molecules at a specific location on a tissue surface. We present the mass spectrometry images of all 15 flavonoids and performed our classification analysis in **Figure 4**.

Ion images of the 15 flavonoids indicated that they can be broadly classified into four types. Four compounds were distributed mainly in the embryo: liquiritigenin (*m/z* 257.081, [M+H]^+^), luteolin (*m/z* 287.056, [M+H]^+^), dihydrokaempferol (*m/z* 289.071, [M+H]^+^), and kaempferol (*m/z* 309.036, [M+Na]^+^). Luteolin was highly concentrated in the embryo and less concentrated in other parts, while kaempferol was distributed at low abundance in the cotyledons and more in the embryo. Myricetin (*m/z* 319.046, [M+H]^+^), baicalin (*m/z* 469.073, [M+Na]^+^), and rutin (*m/z* 649.118, [M+K]^+^) were primarily distributed in the cotyledons. Baicalin and rutin were distributed at the periphery of the cotyledons, and myricetin was distributed to one side of the cotyledon gap. Most of the compounds were distributed in both cotyledons and embryos, including naringenin (*m/z* 273.076, [M+H]^+^), apigenin (*m/z* 271.060, [M+H]^+^), daidzein (*m/z* 293.020, [M+Na]^+^), quercetin (*m/z* 303.051, [M+H]^+^), isorhamnetin (*m/z* 317.066, [M+H]^+^), catechin (m/z 329.043, [M+H]^+^), and quercetin 3-β-d-glucoside (*m/z* 465.102, [M+H]^+^). Naringenin and catechin were concentrated throughout the litchi seed, their distribution being more homogeneous and without obvious tissue specificity. Quercetin, quercetin 3-β-d-glucoside, and apigenin were distributed at the periphery of the cotyledons and in the embryo. The compound daidzein was uniformly distributed, whereas isorhamnetin was more distributed at the apical part of the cotyledons. Finally, the taxifolin (*m/z* 305.065, [M+H]^+^) content was low and mainly distributed in the inner seed testa.

Four compounds were mainly distributed in the embryo: liquiritigenin, luteolin, dihydrokaempferol, and kaempferol. As the embryo is the most important part of the seed in plant development, these flavonoids may provide essential substances for growth and development and improve seed resistance. Luteolin was highly concentrated in the embryo and less concentrated in other parts. Luteolin, through inducing root nodulation, plays an important role in nitrogen metabolism in nitrogen-fixing plants and enhanced plant stress tolerance by promoting its nitrogen enrichment (Peters et al., 1986). Liquiritigenin was also mainly concentrated in the embryo and lesser in the cotyledons close to the embryo. Liquiritigenin increases ultraviolet irradiation, indicating its anti-radiation function (Sun et al., 2012). Dihydrokaempferol and kaempferol were interconvertible; therefore, both had similar distribution characteristics and are distributed in the cotyledons as well as the embryo. Many studies have demonstrated that kaempferol, as a precursor of ubiquitin-ketone (coenzyme Q) biosynthesis, is an atypical node between primary and specialized metabolism (Soubeyrand et al., 2018; Berger et al., 2022). Kaempferol is involved in plant defense and signaling in response to stressful conditions (Soubeyrand et al., 2018; Jan et al., 2022). Dihydrokaempferol is involved in plant growth and development. As a precursor of orange pelargonidin-type anthocyanins, dihydrokaempferol plays a role in flower coloring (Johnson et al., 2001). Liquiritigenin rapidly inactivates the PI3K/AKT/mTOR pathway. *In vivo* studies demonstrated that liquiritigenin can significantly inhibit tumor growth, increase cell autophagy, and accelerate cell apoptosis. In addition, it attenuates the malignant-like biological behaviors in triple-negative breast cancer cells through its induction of autophagy-related apoptosis *via* the PI3K/AKT/mTOR pathway (Ji et al., 2021), decreased DNMT activity, and elevated BRCA1 expression and transcriptional activity (Liang et al., 2021). Dihydrokaempferol has strong anti-inflammatory and antioxidant activities, which can improve the inflammatory performance and oxidative stress state of acute pancreatitis (Liang et al., 2020; Zhang et al., 2021). In contrast, kaempferol shows more pharmacological activities, such as anti-bacterial (Yeon et al., 2019), anti-inflammatory (Yeon et al., 2019), anti-oxidant (Chen and Chen, 2013), antitumor (Calderón-Montaño et al., 2011), and anti-diabetic activities (Yang et al., 2021b), and are cardio-protective (Chen et al., 2022b) and neuro-protective (Wang et al., 2020). Currently, kaempferol is also commonly used in cancer chemotherapy (Ren et al., 2019). The mechanisms of kaempferol’s anticancer include apoptosis, cell cycle arrest at the G2/M phase, downregulation of epithelial–mesenchymal transition-related markers, and repression of overactivation of the phosphatidylinositol 3-kinase/protein kinase B signaling pathway (Imran et al., 2019; Wang et al., 2019). Luteolin sensitizes cancer cells to treatment-induced cytotoxicity *via* suppressing cell survival pathways and enhancing apoptosis pathways, including the apoptosis pathway of the tumor suppressor protein p53 (Lin et al., 2008). These compounds can be extracted from the embryo of litchi seeds, which is convenient for obtaining a higher content of target substances for pharmaceutical and mass production in the future.

Myricetin, baicalin, and rutin were mainly found in the cotyledons of litchi seeds. Myricetin was mainly concentrated on one side of the cotyledon gap, while rutin and baicalin were mainly distributed at the periphery (**Figure 4**). From a physiological point of view, flavonoids such as myricetin and baicalin assist in the reinforcement of plant tissues, maintenance of seed dormancy, and longevity of seeds during storage (Shirley, 1998). Rutin may participate in strengthening the plant’s defense system against environmental stresses, including UV exposure, low-temperature stress, drought stress, and bacterial pathogen infection (Suzuki et al., 2015; Yang et al., 2016). Myricetin has therapeutic effects on a variety of diseases, such as inflammation, cerebral ischemia, Alzheimer’s disease (AD), cancer, diabetes, pathogenic microorganism infection, thrombosis, and atherosclerosis (Song et al., 2021). Furthermore, myricetin has been reported to regulate the expression of STAT3, PI3K/AKT/mTOR, AChE, IκB/NF-κB, BrdU/NeuN, Hippo, eNOS/NO, ACE, MAPK, Nrf2/HO-1, TLR, and GSK-3β (Song et al., 2021). Rutin shows clear antioxidant and anticancer effects, including a strong ability to inhibit tumors in breast cancer, especially triple-negative breast cancer (Iriti et al., 2017; Liang et al., 2021). Baicalin, similar to rutin and myricetin, has inhibitory effects on lung, breast, and bladder cancers, through different signaling pathways and mechanisms (Ge et al., 2021; Kong et al., 2021; Zhao et al., 2021). Owing to their important pharmacological effects, our study of their spatial distribution provided a basis for the precise extraction of flavonoids for developing drugs.

Seven flavonoids, i.e., naringenin, apigenin, daidzein, quercetin, isorhamnetin, catechin, and quercetin-3-β-d-glucoside, were mainly found in both the cotyledon and embryo of litchi seeds. Among these compounds, catechin, naringenin, daidzein, apigenin, and quercetin-3-β-d-glucoside have homogeneous distributions with relatively high abundance. Isorhamnetin was mainly distributed in the radicle and tip of the cotyledon, while quercetin was distributed at the periphery of the cotyledon. Flavonoids are secondary metabolites in plants that play a critical role in impairing ultraviolet irradiation, regulating the oxidative stress response, and influencing the transport of plant hormones, flower coloring, and pathogen resistance (Buer et al., 2010; Chen et al., 2022a). Naringenin plays various roles in plant–microbe interactions (An et al., 2021). Lignin biosynthesis and coenzyme ligase (4CL) are involved in plant growth, and naringenin is one of the metabolites in this pathway that inhibit enzymes such as 4-CL (Deng et al., 2004). Apigenin (4′,5,7-trihydroxyflavone) is a bioactive compound that belongs to the flavone class, and it is the aglycone of many naturally occurring glycosides. It ameliorates the damaging effects of salinity on rice seedlings, presumably by regulating selective ion uptake by roots and translocation to shoots, thus maintaining the higher K^+^/Na^+^ ratio critical for normal plant growth under salinity stress (Mekawy et al., 2018). Daidzein, as an isoflavonoid, plays crucial roles in the expression of the nod genes of rhizobial bacteria. The expression of this compound in roots will increase the synthesis and secretion of nodulation factors, promoting a series of physiological changes in plant cells and initiating the formation of nodules (Bosse et al., 2021). Quercetin promotes a series of physiological and biochemical processes in plants, including seed germination, pollen growth, photosynthesis, and antioxidant machinery, thus facilitating proper plant growth and development (Singh et al., 2021). In addition, quercetin is an antioxidant that enhances plant resistance to some biotic and abiotic stresses. Quercetin-3-β-d-glucoside is a quercetin-derived compound with attached glucose instead of the 3-OH group of quercetin. Isorhamnetin is a methylated flavonoid derived from quercetin. Catechins, as a type of flavonoid, also belong to phenolic compounds. Making up more than 70% of polyphenols, catechins consist of ester and non-ester catechins. The multifunctional catechins contribute to decreased reactive oxygen species and better adaptability of plants to the environment (Jiang et al., 2020). Some of these flavonoids have been previously extracted from litchi seeds, for example, catechin and naringenin (Zhu et al., 2019). Similar to other flavonoids, most of these compounds have many pharmacological effects, including anti-inflammatory, antioxidant, and antidiabetic activities. In particular, since the start of the COVID-19 epidemic, antiviral activity has been reported for catechin (Mishra et al., 2021) and quercetin (Bernini and Velotti, 2021). The antitumor effects of flavonoids have also been extensively studied, with the following mechanisms reported: inducing oxidative stress (Souza et al., 2017), enhancing chemotherapy drug effect (Yang et al., 2021a), and regulating signaling pathways (Amado et al., 2014). Notably, daidzein is a phytohormone similar to estrogens and thus may have a therapeutic effect on estrogen-dependent diseases (Meng et al., 2017). Therefore, flavonoid compounds are useful for developing drug-based therapies, and exploring the distribution of flavonoids will facilitate efficient extraction and utilization.

Although taxifolin was successfully detected in sections *in situ* using MALDI-MSI, the abundance of this compound was low. As shown in **Figure 4**, taxifolin was mainly found in the testa and peripheral part of the cotyledons, indicating that the compound can protect seed embryos from external biotic and abiotic factors, such as soil microbes (e.g., fungi and bacteria) and saline-alkali abiotic stress, thus improving seed vitality and germination rate (Ninfali et al., 2020; Wan et al., 2020). By regulating the aromatic hydrocarbon receptor/cytochrome P450 1A1 (CYP1A1) signaling pathway, taxifolin can significantly inhibit the proliferation, migration, invasion, and viability of gastric cancer cells (Xie et al., 2021). Similarly, the same effect of taxifolin has been observed on breast cancer by promoting mesenchymal-to-epithelial transition (EMT) through β-catenin signaling (Von Minckwitz et al., 2019).

## Conclusion

MALDI-MSI was used for *in situ* detection and imaging of flavonoid distribution in litchi seeds for the first time. Overall, 15 flavonoids were successfully imaged. Among them, four (dihydrokaempferol, liquiritigenin, luteolin, and kaempferol) were distributed in the seed embryo, three (rutin, baicalin, and myricetin) were mainly found in the cotyledons, seven (quercetin, naringenin, isorhamnetin, daidzein, apigenin, catechin, and quercetin 3-β-d-glucoside) were enriched in both the embryo and cotyledons, and one (taxifolin) was mainly detected in the inner testa. Our MALDI-MSI results showed clear tissue distribution heterogeneity for the different flavonoid compounds in litchi seeds. Such information will be important for further study to understand the physiological and chemical functions of such flavonoid compounds. Furthermore, our study provides a basis for further improving the efficiency of extracting and utilizing bioactive compounds from litchi seeds.

## Data availability statement

The original contributions presented in the study are included in the article/**Supplementary Material**. Further inquiries can be directed to the corresponding author.

## Author contributions

Concepts: YL and XN. Design: ZW, FJ, and YL. Literature search: JW, ZZ, and YL. Data acquisition and analysis: ZW, XN, YL, and ZZ. Writing—original draft: YL, XN, and JW. Writing—review and editing: FJ, ZW, YL, and XN. Funding acquisition: YL, XN, and JW. Supervision: FJ and ZW. All authors contributed to the article and approved the submitted version.

## References

[B1] Amado N. G. Predes D. Fonseca B. F. Cerqueira D. M. Reis A. H. Dudenhoeffer A. C.. (2014). Isoquercitrin suppresses colon cancer cell growth *in vitro* by targeting the wnt/β-catenin signaling pathway. J. Biol. Chem. 289, 35456–35467. doi: 10.1074/jbc.M114.621599 25359775 PMC4271231

[B2] An J. Kim S. H. Bahk S. Vuong U. T. Nguyen N. T. Do H. L.. (2021). Naringenin induces pathogen resistance against pseudomonas syringae through the activation of NPR1 in arabidopsis. Front. Plant Sci. 12. doi: 10.3389/fpls.2021.672552 PMC8173199 34093630

[B3] Berger A. Latimer S. Stutts L. R. Soubeyrand E. Block A. K. Basset G. J. (2022). Kaempferol as a precursor for ubiquinone (coenzyme q) biosynthesis: An atypical node between specialized metabolism and primary metabolism. Curr. Opin. Plant Biol. 66, 102165. doi: 10.1016/j.pbi.2021.102165 35026487

[B4] Berk Ş. Kaya S. Akkol E. K. Bardakçı H. (2022). A comprehensive and current review on the role of flavonoids in lung cancer-experimental and theoretical approaches. Phytomedicine 98, 153938. doi: 10.1016/j.phymed.2022.153938 35123170

[B5] Bernini R. Velotti F. (2021). Natural polyphenols as immunomodulators to rescue immune response homeostasis: Quercetin as a research model against severe COVID-19. Molecules 26. doi: 10.3390/molecules26195803 PMC8510228 34641348

[B6] Bosse M. A. Silva M. B. D. Oliveira N. Araujo M. A. Rodrigues C. Azevedo J. P.. (2021). Physiological impact of flavonoids on nodulation and ureide metabolism in legume plants. Plant Physiol. Biochem. 166, 512–521. doi: 10.1016/j.plaphy.2021.06.007 34171572

[B7] Buer C. S. Imin N. Djordjevic M. A. (2010). Flavonoids: New roles for old molecules. J. Integr. Plant Biol. 52, 98–111. doi: 10.1111/j.1744-7909.2010.00905.x 20074144

[B8] Calderón-Montaño J. M. Burgos-Morón E. Pérez-Guerrero C. López-Lázaro M. (2011). A review on the dietary flavonoid kaempferol. Mini Rev. Med. Chem. 11, 298–344. doi: 10.2174/138955711795305335 21428901

[B9] Casadonte R. Caprioli R.M. (2011). Proteomic analysis of formalin-fixed paraffin-embedded tissue by MALDI imaging mass spectrometry. Nat. Protoc. 6, 1695–1709. doi: 10.1038/nprot.2011.388 22011652 PMC3516368

[B10] Chen A. Y. Chen Y. C. (2013). A review of the dietary flavonoid, kaempferol on human health and cancer chemoprevention. Food Chem. 138, 2099–2107. doi: 10.1016/j.foodchem.2012.11.139 23497863 PMC3601579

[B11] Chen J. H. Hou N. Xv X. Zhang D. Fan T. Q. Zhang Q. X.. (2022a). Flavonoid synthesis and metabolism during the fruit development in hickory (Carya cathayensis). Front. Plant Sci. 13. doi: 10.3389/fpls.2022.896421 PMC9125235 35615140

[B12] Chen M. Xiao J. El-Seedi H. R. Woźniak K. S. Daglia M. Little P. J.. (2022b). Kaempferol and atherosclerosis: From mechanism to medicine. Crit. Rev. Food Sci. Nutr. 1-19. doi: 10.1080/10408398.2022.2121261 36099317

[B13] Choi S.-A. Lee J. E. Kyung M. J. Youn J. H. Oh J. B. Whang W. K. (2017). Anti-diabetic functional food with wasted litchi seed and standard of quality control. Appl. Biol. Chem. 60, 197–204. doi: 10.1007/s13765-017-0269-9

[B14] Ciumărnean L. Milaciu M. V. Runcan O. Vesa Ș C. Răchișan A. L. Negrean V.. (2020). The effects of flavonoids in cardiovascular diseases. Molecules 25. doi: 10.3390/molecules25184320 PMC7571023 32967119

[B15] Deng F. Aoki M. Yogo Y. (2004). Effect of naringenin on the growth and lignin biosynthesis of gramineous plants. Weed Biol. Manage. 4, 49–55. doi: 10.1111/j.1445-6664.2003.00119.x

[B16] Dong X. Huang Y. Wang Y. He X. (2019). Anti-inflammatory and antioxidant jasmonates and flavonoids from lychee seeds. J. Funct. Foods 54, 74–80. doi: 10.1016/j.jff.2018.12.040

[B17] Emanuele S. Lauricella M. Calvaruso G. D'anneo A. Giuliano M. (2017). Litchi chinensis as a functional food and a source of antitumor compounds: An overview and a description of biochemical pathways. Nutrients 9, 992. doi: 10.3390/nu9090992 28885570 PMC5622752

[B18] Fan X. Bai J. Zhao S. Hu M. Sun Y. Wang B.. (2019). Evaluation of inhibitory effects of flavonoids on breast cancer resistance protein (BCRP): From library screening to biological evaluation to structure-activity relationship. Toxicol. In Vitro 61, 104642. doi: 10.1016/j.tiv.2019.104642 31493543

[B19] Ge A. Liu L. Deng X. Luo J. Xu Y. (2021). Exploring the mechanism of baicalin intervention in breast cancer based on MicroRNA microarrays and bioinformatics strategies. Evid. Based Complement. Alternat. Med. 2021, 7624415. doi: 10.1155/2021/7624415 34966436 PMC8712139

[B20] Guo Y. Ding S. J. Ding X. Liu Z. Wang J. L. Chen Y.. (2022). Effects of selected flavonoids oncellproliferation and differentiation of porcine muscle stem cells for cultured meat production. Food Res. Int. 160, 111459. doi: 10.1016/j.foodres.2022.111459 36076368

[B21] Hu F.-C. Chen Z. Wang X.-H. Wang J.-B. Fan H.-Y. Qin Y.-H.. (2021). Construction of high-density SNP genetic maps and QTL mapping for dwarf-related traits in litchi chinensis sonn. J. Integr. Agr. 20, 2900–2913. doi: 10.1016/s2095-3119(20)63387-1

[B22] Huang L. Tang X. Zhang W. Jiang R. Chen D. Zhang J.. (2016). Imaging of endogenous metabolites of plant leaves by mass spectrometry based on laser activated electron tunneling. Sci. Rep. 6, 24164. doi: 10.1038/srep24164 27053227 PMC4823709

[B23] Imran M. Salehi B. Sharifi-Rad J. Aslam Gondal T. Saeed F. Imran A.. (2019). Kaempferol: A key emphasis to its anticancer potential. Molecules 24. doi: 10.3390/molecules24122277 PMC6631472 31248102

[B24] Iriti M. Kubina R. Cochis A. Sorrentino R. Varoni E. M. Kabała-Dzik A.. (2017). Rutin, a quercetin glycoside, restores chemosensitivity in human breast cancer cells. Phytother. Res. 31, 1529–1538. doi: 10.1002/ptr.5878 28752532

[B25] Jan R. Khan M. Asaf S. Lubna Asif S. Kim K. M. (2022). Bioactivity and therapeutic potential of kaempferol and quercetin: New insights for plant and human health. Plants (Basel) 11. doi: 10.3390/plants11192623 PMC9571405 36235488

[B26] Ji Y. Hu W. Jin Y. Yu H. Fang J. (2021). Liquiritigenin exerts the anti-cancer role in oral cancer *via* inducing autophagy-related apoptosis through PI3K/AKT/mTOR pathway inhibition *in vitro* and *in vivo* . Bioengineered 12, 6070–6082. doi: 10.1080/21655979.2021.1971501 34488535 PMC8806794

[B27] Jiang C. K. Ma J. Q. Liu Y. F. Chen J. D. Ni D. J. Chen L. (2020). Identification and distribution of a single nucleotide polymorphism responsible for the catechin content in tea plants. Hortic. Res. 7, 24. doi: 10.1038/s41438-020-0247-y 32140233 PMC7049304

[B28] Johnson E. T. Ryu S. Yi H. Shin B. Cheong H. Choi G. (2001). Alteration of a single amino acid changes the substrate specificity of dihydroflavonol 4-reductase. Plant J. 25, 325–333. doi: 10.1046/j.1365-313x.2001.00962.x 11208024

[B29] Khan H. Ullah H. Martorell M. Valdes S. E. Belwal T. Tejada S.. (2021). Flavonoids nanoparticles in cancer: Treatment, prevention and clinical prospects. Semin. Cancer Biol. 69, 200–211. doi: 10.1016/j.semcancer.2019.07.023 31374244

[B30] Kong N. Chen X. Feng J. Duan T. Liu S. Sun X.. (2021). Baicalin induces ferroptosis in bladder cancer cells by downregulating FTH1. Acta Pharm. Sin. B. 11, 4045–4054. doi: 10.1016/j.apsb.2021.03.036 35024325 PMC8727776

[B31] Li H. Huang D. Ma Q. Qi W. Li H. (2020). Factors influencing the technology adoption behaviours of litchi farmers in China. Sustainability 12, 271. doi: 10.3390/su12010271

[B32] Li B. Neumann E. K. Ge J. Gao W. Yang H. Li P.. (2018a). Interrogation of spatial metabolome of ginkgo biloba with high-resolution matrix-assisted laser desorption/ionization and laser desorption/ionization mass spectrometry imaging. Plant Cell Environ. 41, 2693–2703. doi: 10.1111/pce.13395 29966033

[B33] Li Y. Zhang T. Chen G. Y. (2018b). Flavonoids and colorectal cancer prevention. Antioxidants (Basel) 7. doi: 10.3390/antiox7120187 PMC6316869 30544686

[B34] Liang X. Hu C. Liu C. Yu K. Zhang J. Jia Y. (2020). Dihydrokaempferol (DHK) ameliorates severe acute pancreatitis (SAP) *via* Keap1/Nrf2 pathway. Life Sci. 261, 118340. doi: 10.1016/j.lfs.2020.118340 32860805

[B35] Liang F. Zhang H. Gao H. Cheng D. Zhang N. Du J.. (2021). Liquiritigenin decreases tumorigenesis by inhibiting DNMT activity and increasing BRCA1 transcriptional activity in triple-negative breast cancer. Exp. Biol. Med. (Maywood) 246, 459–466. doi: 10.1177/1535370220957255 32938226 PMC7885050

[B36] Lin Y. Shi R. Wang X. Shen H. M. (2008). Luteolin, a flavonoid with potential for cancer prevention and therapy. Curr. Cancer Drug Targets 8, 634–646. doi: 10.2174/156800908786241050 18991571 PMC2615542

[B37] Liskova A. Samec M. Koklesova L. Brockmueller A. Zhai K. Abdellatif B.. (2021). Flavonoids as an effective sensitizer for anti-cancer therapy: Insights into multi-faceted mechanisms and applicability towards individualized patient profiles. Epma. J. 12, 155–176. doi: 10.1007/s13167-021-00242-5 34025826 PMC8126506

[B38] Magne Nde C. B. Zingue S. Winter E. Creczynski-Pasa T. B. Michel T. Fernandez X.. (2015). Flavonoids, breast cancer chemopreventive and/or chemotherapeutic agents. Curr. Med. Chem. 22, 3434–3446. doi: 10.2174/0929867322666150729115321 26219391

[B39] Maleki S. J. Crespo J. F. Cabanillas B. (2019). Anti-inflammatory effects of flavonoids. Food Chem. 299, 125124. doi: 10.1016/j.foodchem.2019.125124 31288163

[B40] Martinez-Perez C. Ward C. Cook G. Mullen P. Mcphail D. Harrison D. J.. (2014). Novel flavonoids as anti-cancer agents: mechanisms of action and promise for their potential application in breast cancer. Biochem. Soc. Trans. 42, 1017–1023. doi: 10.1042/BST20140073 25109996

[B41] Mekawy A. M. M. Abdelaziz M. N. Ueda A. (2018). Apigenin pretreatment enhances growth and salinity tolerance of rice seedlings. Plant Physiol. Biochem. 130, 94–104. doi: 10.1016/j.plaphy.2018.06.036 29980098

[B42] Meng H. Fu G. Shen J. Shen K. Xu Z. Wang Y.. (2017). Ameliorative effect of daidzein on cisplatin-induced nephrotoxicity in mice *via* modulation of inflammation, oxidative stress, and cell death. Oxid. Med. Cell. Longev. 2017, 3140680. doi: 10.1155/2017/3140680 28831294 PMC5558675

[B43] Miron A. Aprotosoaie A. C. Trifan A. Xiao J. (2017). Flavonoids as modulators of metabolic enzymes and drug transporters. Ann. N. Y. Acad. Sci. 1398, 152–167. doi: 10.1111/nyas.13384 28632894

[B44] Mishra C. B. Pandey P. Sharma R. D. Malik M. Z. Mongre R. K. Lynn A. M.. (2021). Identifying the natural polyphenol catechin as a multi-targeted agent against SARS-CoV-2 for the plausible therapy of COVID-19: An integrated computational approach. Brief Bioinform. 22, 1346–1360. doi: 10.1093/bib/bbaa378 33386025 PMC7799228

[B45] Ninfali P. Panato A. Bortolotti F. Valentini L. Gobbi P. (2020). Morphological analysis of the seeds of three pseudocereals by using light microscopy and ESEM-EDS. Eur. J. Histochem. 64. doi: 10.4081/ejh.2020.3075 PMC6985911 31941265

[B46] Pan H. Zhou W. He W. Liu X. Ding Q. Ling L.. (2012). Genistein inhibits MDA-MB-231 triple-negative breast cancer cell growth by inhibiting NF-kappaB activity *via* the notch-1 pathway. Int. J. Mol. Med. 30, 337–343. doi: 10.3892/ijmm.2012.990 22580499

[B47] Park K. I. Park H. S. Nagappan A. Hong G. E. Lee D. H. Kang S. R.. (2012). Induction of the cell cycle arrest and apoptosis by flavonoids isolated from Korean citrus aurantium l. @ in non-small-cell lung cancer cells. Food Chem. 135, 2728–2735. doi: 10.1016/j.foodchem.2012.06.097 22980865

[B48] Peluso I. Raguzzini A. Serafini M. (2013). Effect of flavonoids on circulating levels of TNF-alpha and IL-6 in humans: a systematic review and meta-analysis. Mol. Nutr. Food Res. 57, 784–801. doi: 10.1002/mnfr.201200721 23471810

[B49] Peters N. K. Frost J. W. Long S. R. (1986). A plant flavone, luteolin, induces expression of rhizobium meliloti nodulation genes. Science 233, 977–980. doi: 10.1126/science.3738520 3738520

[B50] Piehowski P. D. Zhu Y. Bramer L. M. Stratton K. G. Zhao R. Orton D. J.. (2020). Automated mass spectrometry imaging of over 2000 proteins from tissue sections at 100-mum spatial resolution. Nat. Commun. 11, 8. doi: 10.1038/s41467-019-13858-z 31911630 PMC6946663

[B51] Punia S. Kumar M. (2021). Litchi (Litchi chinenis) seed: Nutritional profile, bioactivities, and its industrial applications. Trends Food Sci. Technol. 108, 58–70. doi: 10.1016/j.tifs.2020.12.005

[B52] Qin L. Zhang Y. Liu Y. He H. Han M. Li Y.. (2018). Recent advances in matrix-assisted laser desorption/ionisation mass spectrometry imaging (MALDI-MSI) for *in situ* analysis of endogenous molecules in plants. Phytochem. Anal. 29, 351–364. doi: 10.1002/pca.2759 29667236

[B53] Rakha A. Umar N. Rabail R. Butt M. S. Kieliszek M. Hassoun A.. (2022). Anti-inflammatory and anti-allergic potential of dietary flavonoids: A review. Biomed. Pharmacother. 156, 113945. doi: 10.1016/j.biopha.2022.113945 36411631

[B54] Ren J. Lu Y. Qian Y. Chen B. Wu T. Ji G. (2019). Recent progress regarding kaempferol for the treatment of various diseases (Review). Exp. Ther. Med. 18, 2759–2776. doi: 10.3892/etm.2019.7886 31572524 PMC6755486

[B55] Shi Y. Hu H. Hao Q. Wu R. Wang L. Qin L.. (2022). Michler's ethylketone as a novel negative-ion matrix for the enhancement of lipid MALDI tissue imaging. Chem. Commun. (Camb.) 58, 633–636. doi: 10.1039/d1cc05718a 34897326

[B56] Shirley B. W. (1998). Flavonoids in seeds and grains: Physiological function, agronomic importance and the genetics of biosynthesis. Seed Sci. Res. 8, 415–422. doi: 10.1017/s0960258500004372

[B57] Singh P. Arif Y. Bajguz A. Hayat S. (2021). The role of quercetin in plants. Plant Physiol. Biochem. 166, 10–19. doi: 10.1016/j.plaphy.2021.05.023 34087741

[B58] Song X. Tan L. Wang M. Ren C. Guo C. Yang B.. (2021). Myricetin: A review of the most recent research. Biomed. Pharmacother. 134, 111017. doi: 10.1016/j.biopha.2020.111017 33338751

[B59] Soubeyrand E. Johnson T. S. Latimer S. Block A. Kim J. Colquhoun T. A.. (2018). The peroxidative cleavage of kaempferol contributes to the biosynthesis of the benzenoid moiety of ubiquinone in plants. Plant Cell 30, 2910–2921. doi: 10.1105/tpc.18.00688 30429224 PMC6354277

[B60] Souza R. P. Bonfim-Mendonca P. S. Gimenes F. Ratti B. A. Kaplum V. Bruschi M. L.. (2017). Oxidative stress triggered by apigenin induces apoptosis in a comprehensive panel of human cervical cancer-derived cell lines. Oxid. Med. Cell. Longev. 2017, 1512745. doi: 10.1155/2017/1512745 28191273 PMC5278229

[B61] Sudhakaran M. Sardesai S. Doseff A. I. (2019). Flavonoids: New frontier for immuno-regulation and breast cancer control. Antioxidants (Basel) 8. doi: 10.3390/antiox8040103 PMC6523469 30995775

[B62] Sun R. Hikosaka S. Goto E. Sawada H. Saito T. Kudo T.. (2012). Effects of UV irradiation on plant growth and concentrations of four medicinal ingredients in Chinese licorice (Glycyrrhiza uralensis). Acta Hortic. 956, 643–648. doi: 10.17660/ActaHortic.2012.956.77

[B63] Surichan S. Androutsopoulos V. P. Sifakis S. Koutala E. Tsatsakis A. Arroo R. R.. (2012). Bioactivation of the citrus flavonoid nobiletin by CYP1 enzymes in MCF7 breast adenocarcinoma cells. Food Chem. Toxicol. 50, 3320–3328. doi: 10.1016/j.fct.2012.06.030 22743247

[B64] Suzuki T. Morishita T. Kim S.-J. Park S.-U. Woo S.-H. Noda T.. (2015). Physiological roles of rutin in the buckwheat plant. Japan Agric. Res. Quarterly: JARQ 49, 37–43. doi: 10.6090/jarq.49.37

[B65] Taira S. Tokai M. Kaneko D. Katano H. Kawamura-Konishi Y. (2015). Mass spectrometry imaging analysis of location of procymidone in cucumber samples. J. Agric. Food Chem. 63, 6109–6112. doi: 10.1021/acs.jafc.5b00957 25943531

[B66] Tang Y. Yu C. Wu J. Chen H. Zeng Y. Wang X.. (2018). Lychee seed extract protects against neuronal injury and improves cognitive function in rats with type II diabetes mellitus with cognitive impairment. Int. J. Mol. Med. 41, 251–263. doi: 10.3892/ijmm.2017.3245 29138799 PMC5746317

[B67] Tautenhahn R. Cho K. Uritboonthai W. Zhu Z. Patti G. J. Siuzdak G. (2012). An accelerated workflow for untargeted metabolomics using the METLIN database. Nat. Biotechnol. 30, 826–828. doi: 10.1038/nbt.2348 PMC3666346 22965049

[B68] Van De Plas R. Yang J. Spraggins J. Caprioli R. M. (2015). Image fusion of mass spectrometry and microscopy: A multimodality paradigm for molecular tissue mapping. Nat. Methods 12, 366–372. doi: 10.1038/nmeth.3296 25707028 PMC4382398

[B69] Von Minckwitz G. Huang C. S. Mano M. S. Loibl S. Mamounas E. P. Untch M.. (2019). Trastuzumab emtansine for residual invasive HER2-positive breast cancer. N. Engl. J. Med. 380, 617–628. doi: 10.1056/NEJMoa1814017 30516102

[B70] Vue B. Zhang S. Chen Q. H. (2016). Flavonoids with therapeutic potential in prostate cancer. Anticancer Agents Med. Chem. 16, 1205–1229. doi: 10.2174/1871520615666151008122622 26446382

[B71] Wan L. Lei Y. Yan L. Liu Y. Pandey M. K. Wan X.. (2020). Transcriptome and metabolome reveal redirection of flavonoids in a white testa peanut mutant. BMC Plant Biol. 20, 161. doi: 10.1186/s12870-020-02383-7 32293272 PMC7161308

[B72] Wang J. Mao J. Wang R. Li S. Wu B. Yuan Y. (2020). Kaempferol protects against cerebral ischemia reperfusion injury through intervening oxidative and inflammatory stress induced apoptosis. Front. Pharmacol. 11. doi: 10.3389/fphar.2020.00424 PMC7174640 32351385

[B73] Wang X. Yang Y. An Y. Fang G. (2019). The mechanism of anticancer action and potential clinical use of kaempferol in the treatment of breast cancer. Biomed. Pharmacother. 117, 109086. doi: 10.1016/j.biopha.2019.109086 31200254

[B74] Wishart D. S. Guo A. Oler E. Wang F. Anjum A. Peters H.. (2022). HMDB 5.0: The human metabolome database for 2022. Nucleic Acids Res. 50, D622–D631. doi: 10.1093/nar/gkab1062 34986597 PMC8728138

[B75] Wu R. Qin L. Chen L. Ma R. Chen D. Liu H.. (2021). Copper adhesive tape attached to the reverse side of a non-conductive glass slide to achieve protein MALDI-imaging in FFPE-tissue sections. Chem. Commun. (Camb.) 57, 10707–10710. doi: 10.1039/d1cc03629g 34542115

[B76] Xie J. Pang Y. Wu X. (2021). Taxifolin suppresses the malignant progression of gastric cancer by regulating the AhR/CYP1A1 signaling pathway. Int. J. Mol. Med. 48. doi: 10.3892/ijmm.2021.5030 PMC8448545 34490474

[B77] Yang L. Gao Y. Bajpai V. K. El-Kammar H. A. Simal-Gandara J. Cao H.. (2021b). Advance toward isolation, extraction, metabolism and health benefits of kaempferol, a major dietary flavonoid with future perspectives. Crit. Rev. Food Sci. Nutr. 1–17. doi: 10.1080/10408398.2021.1980762 34554029

[B78] Yang C. Song J. Hwang S. Choi J. Song G. Lim W. (2021a). Apigenin enhances apoptosis induction by 5-fluorouracil through regulation of thymidylate synthase in colorectal cancer cells. Redox Biol. 47, 102144. doi: 10.1016/j.redox.2021.102144 34562873 PMC8476449

[B79] Yang W. Xu X. Li Y. Wang Y. Li M. Wang Y.. (2016). Rutin-mediated priming of plant resistance to three bacterial pathogens initiating the early SA signal pathway. PLos One 11, e0146910. doi: 10.1371/journal.pone.0146910 26751786 PMC4713477

[B80] Yao P. Gao Y. Simal-Gandara J. Farag M. A. Chen W. Yao D.. (2021). Litchi (Litchi chinensis sonn.): A comprehensive review of phytochemistry, medicinal properties, and product development. Food Funct. 12, 9527–9548. doi: 10.1039/d1fo01148k 34664581

[B81] Yeon M. J. Lee M. H. Kim D. H. Yang J. Y. Woo H. J. Kwon H. J.. (2019). Anti-inflammatory effects of kaempferol on helicobacter pylori-induced inflammation. Biosci. Biotechnol. Biochem. 83, 166–173. doi: 10.1080/09168451.2018.1528140 30286691

[B82] Zaima N. Goto-Inoue N. Hayasaka T. Setou M. (2010). Application of imaging mass spectrometry for the analysis of oryza sativa rice. Rapid Commun. Mass Spectrom. 24, 2723–2729. doi: 10.1002/rcm.4693 20814978

[B83] Zhang H. W. Hu J. J. Fu R. Q. Liu X. Zhang Y. H. Li J.. (2018). Flavonoids inhibit cell proliferation and induce apoptosis and autophagy through downregulation of PI3Kγ mediated PI3K/AKT/mTOR/p70S6K/ULK signaling pathway in human breast cancer cells. Sci. Rep. 8, 11255. doi: 10.1038/s41598-018-29308-7 30050147 PMC6062549

[B84] Zhang J. Hu C. Li X. Liang L. Zhang M. Chen B.. (2021). Protective effect of dihydrokaempferol on acetaminophen-induced liver injury by activating the SIRT1 pathway. Am. J. Chin. Med. 49, 705–718. doi: 10.1142/S0192415X21500324 33657990

[B85] Zhao L. Wang K. Wang K. Zhu J. Hu Z. (2020). Nutrient components, health benefits, and safety of litchi (Litchi chinensis sonn.): A review. Compr. Rev. Food Sci. Food Saf. 19, 2139–2163. doi: 10.1111/1541-4337.12590 33337091

[B86] Zhao F. Zhao Z. Han Y. Li S. Liu C. Jia K. (2021). Baicalin suppresses lung cancer growth phenotypes *via* miR-340-5p/NET1 axis. Bioengineered 12, 1699–1707. doi: 10.1080/21655979.2021.1922052 33955315 PMC8806212

[B87] Zhu X. Wang H. Sun J. Yang B. Duan X. Jiang Y. (2019). Pericarp and seed of litchi and longan fruits: Constituent, extraction, bioactive activity, and potential utilization. J. Zhejiang Univ. Sci. B 20, 503–512. doi: 10.1631/jzus.B1900161 31090276 PMC6568221

