# Supplementary material for: Visualizing the distribution of flavonoids in litchi (Litchi chinenis) seeds through matrix-assisted laser desorption/ionization mass spectrometry imaging
Source: Front Plant Sci. 2023 Feb 24;14:1144449. doi: 10.3389/fpls.2023.1144449 (PMC9998689; doi:10.3389/fpls.2023.1144449)
Supplement: Supplementary file 1 [file DataSheet_1.pdf]

Supplementary Materials

Visualizing the distribution of flavonoids in litchi (*Litchi chinenis*) seed through matrix-assisted laser desorption/ionization mass spectrometry imaging

Yukun Liu[1,#], Xiaofei Nei[2,#], Jilong Wang[3], Zhenqi Zhao[4], Zhimei Wang[5], and Fang Ju[2,*]

1. Department of Breast Surgery, Breast Disease Center, Affiliated Qingdao Central Hospital, Qingdao University, Qingdao 266042, China.
2. Department of Oncology, Affiliated Qingdao Central Hospital, Qingdao University, Qingdao 266042, China.
3. Department of Acupuncture and Moxibustion, Affiliated Qingdao Central Hospital, Qingdao University, Qingdao 266042, China.
4. Department of Radiology, Affiliated Qingdao Central Hospital, Qingdao University, Qingdao 266042, China.
5. Department of Gynecological Neoplasms, Affiliated Qingdao Central Hospital, Qingdao University, Qingdao 266042, China.

[#]These authors contributed equally to this work.

***Corresponding author:**

Prof. Fang Ju, Ph.D.

Department of Oncology, Affiliated Qingdao Central Hospital, Qingdao University

#127 Siliu South Road, Shibei District

Qingdao, 266042, China

Email: 291838679@qq. com

Tel.: +86-532-84961787; **Fax**: +86-532-84961787

Supplementary Materials--METHODS

Optimization of Matrix Solution Composition. Orthogonal-array testing with three variables, including 2-MBT concentration, percent methanol, and percent TFA, was performed to establish the optimal 2-MBT matrix solution. Solution compositions of 70, 80, and 90% methanol in water; 2-MBT concentrations of 10, 15, and 20 mg/mL; and 0.1, 0.2, and 0.3%TFA were chosen for the matrix optimization. Serial (12 μm thick) homogeneous rat liver tissue sections were used for this optimization.

Flavonoids Extraction. Total flavonoids were extracted from a *ca.* 250-mg aliquot of the litchi seed tissue using an ultrasonic–microwave synergistic extraction protocol previously described (Chaves et al., 2020; Li et al., 2021). Briefly, the tissue was homogenized with 1.0-mL water in a 2.0-mL Eppendorf tube with the aid of two 5-mm stainless steel balls at a vibrating frequency of 30 Hz for 30 s $\times$3 on a Retsch MM400 mixer mill (Haan, Germany). The homogenized litchi seed tissue and supernatant were transferred to a new 2.0-mL Eppendorf tube and then dried in the Savant SPD1010 SpeedVac concentrator. Next, an ultrasonic-microwave synergistic extraction apparatus (CW-2000, Shanghai Xintuo Microwave Instrument Co. Ltd., China) equipped with an ultrasonic control device (power from 10 to 800 W) and a microwave control device (power 50 W, frequency 40 kHz) was used for total flavonoid extraction. The dried litchi seed tissue powder was placed in a quartz extraction cell equipped with reflux system. After the apparatus was turned on, extraction time was counted and the extraction was carried out continuously at the preset parameters. When the extraction was completed, and the extracts were collected, filtrated, and fixed for the further determination of total flavonoid content. After the analysis, the remaining extract

was concentrated, freeze-dried, and stored at −20°C. For LC-MS/MS analysis, the freeze-dried total flavonoids were reconstituted in 500 μL of 50% methanol, and 5-μL aliquots were injected.

LC-MS/MS Data Acquisition. A Waters ACQUITY UPLC system coupled online to an LTQ Orbitrap Velos Pro mass spectrometer (Thermo Fisher Scientific, Bremen, Germany), equipped with an atmospheric pressure ESI source was used for LC-MS/MS of flavonoids as a complementary technique for structural confirmation. The mobile phase was 0.01% formic acid in water (solvent A) and 0.01% formic acid in ACN/isopropanol (1:1) (solvent B) for binary gradient elution. The total flavonoids were separated on a Waters BEH C-18 column (2.1 mm ID $\times$ 50 mm length, 1.7 μm particle size) with an elution gradient of 5% to 45% B in 5 min; 45% to 100% B in 15 min and 100% B for 2 min. The column was then equilibrated at 5% B for 3 min before the next injection. The flow rate was 0.35 mL/min, and the column temperature was 45 ℃. LC-MS survey scan data was acquired within a mass range of *m/z* 50 to 1000 and in the FTMS detection mode at a mass resolution of 15,000 FWHM (*m/z* 400). The top 5 most abundant ions in the survey scan were selected for subsequent MS/MS scans using collision-induced dissociation (CID) with normalized collision energy of 30%. The automatic gain control was 2×10^5 ion counts for each ion injection in the ion trap, and the ion injection time limit was 100 ms. During the MS/MS data acquisitions, dynamic exclusion was applied with an ion exclusion time of 15 s.

MS/MS Spectral Analysis. Assignment of the flavonoids was performed by comparing the acquired MS/MS spectra with those in the standard MS/MS libraries of the METLIN

(Tautenhahn et al., 2012) and HMDB (Wishart et al., 2022) database, with the aid of some manual spectral interpretations.

Supplementary Material--FIGURE

A

Components	Concentrations		
Methanol(%)	90 (A1)	80 (A2)	70 (A3)
TFA(%)	0.1 (B1)	0.2 (B2)	0.3 (B3)
2-MBT(mg/mL)	10 (C1)	15(C2)	20(C3)

B

1 A1B1C1	155	164	A1
2 A1B2C2	134		
3 A3B3C3	202		
4 A2B1C2	216	226	A2
5 A2B2C3	235		
6 A2B3C1	227		
7 A3B1C3	178	185	A3
8 A3B2C1	193		
9 A3B3C2	184		

1 A1B1C1	235	183	B1
4 A2B1C2	140		
7 A3B1C3	175		
2 A1B2C2	225	205	B2
5 A2B2C3	221		
8 A3B2C1	168		
3 A1B3C3	210	177	B3
6 A2B3C1	158		
9 A3B3C2	164		

1 A1B1C1	231	181	C1
6 A2B3C1	152		
8 A3B2C1	161		
2 A1B2C2	248	208	C2
4 A2B1C2	216		
9 A3B3C2	160		
3 A1B3C3	204	169	C3
5 A2B2C3	133		
7 A3B1C3	169		

15mg/mL 2-MBT in 80% Methanol containing 0.2% TFA

Supplementary Information Figure S1. Orthogonal array testing for the optimization of 2-MBT matrix solution compositions. Rat-liver tissue sections (n=3) were used for endogenous low-MW compounds detection by MALDI-TOF MS using 2-MBT as a matrix. The numbers of detected ion signals were the average numbers from nine times independent detection results (n=3×3).

REFERENCES

Chaves, J.O., De Souza, M.C., Da Silva, L.C., Lachos-Perez, D., Torres-Mayanga, P.C., Machado, A., Forster-Carneiro, T., Vazquez-Espinosa, M., Gonzalez-De-Peredo, A.V., Barbero, G.F., and Rostagno, M.A. (2020). Extraction of Flavonoids From Natural Sources Using Modern Techniques. *Front. Chem.* 8**,** 507887. doi: 10.3389/fchem.2020.507887

Li, C., Chen, S., Sha, J., Cui, J., He, J., Fu, J., and Shen, Y. (2021). Extraction and purification of total flavonoids from Eupatorium lindleyanum DC. and evaluation of their antioxidant and enzyme inhibitory activities. *Food Sci. Nutr.* 9**,** 2349-2363. doi: 10.1002/fsn3.1999

Tautenhahn, R., Cho, K., Uritboonthai, W., Zhu, Z., Patti, G.J., and Siuzdak, G. (2012). An accelerated workflow for untargeted metabolomics using the METLIN database. *Nat. Biotechnol.* 30**,** 826-828. doi: 10.1038/nbt.2348

Wishart, D.S., Guo, A., Oler, E., Wang, F., Anjum, A., Peters, H., Dizon, R., Sayeeda, Z., Tian, S., Lee, B.L., Berjanskii, M., Mah, R., Yamamoto, M., Jovel, J., Torres-Calzada, C., Hiebert-Giesbrecht, M., Lui, V.W., Varshavi, D., Varshavi, D., Allen, D., Arndt, D., Khetarpal, N., Sivakumaran, A., Harford, K., Sanford, S., Yee, K., Cao, X., Budinski, Z., Liigand, J., Zhang, L., Zheng, J., Mandal, R., Karu, N., Dambrova, M., Schioth, H.B., Greiner, R., and Gautam, V. (2022). HMDB 5.0: the Human Metabolome Database for 2022. *Nucleic Acids Res.* 50**,** D622-D631. doi: 10.1093/nar/gkab1062
